# Supplementary material for: 5p and 3p Strands of miR-34 Family Members Have Differential Effects in Cell Proliferation, Migration, and Invasion in Cervical Cancer Cells
Source: Int J Mol Sci. 2019 Jan 28;20(3):545. doi: 10.3390/ijms20030545 (PMC6387060; doi:10.3390/ijms20030545)
Supplement: Supplementary file 1 [file ijms-20-00545-s001.pdf]

**miRNA Expression and function**	**miRNA analysis methodology**	**Pathology and Human Tissue**	**Cell line**	**Human Tissue**	**Cell process analyzed**	**Targets and methodology used**
**miR-34a-5p** (-)	qRT-PCR	Lung cancer, Colorectal cancer	H1299, NSCLC, BRC, CRC, and A549	NA	Migration Invasion	Axl (Luciferase reporter assay, Western blotting, and RT-PCR)
**miR-34a-5p** (-) **miR-34b-5p** (-) **miR-34c-5p** (-)	qRT-PCR	Gastric cancer	Kato III, AGS, N87, and MKN45	NA	Cell cycle Apoptosis Tumorsphere formation	BCL-2 (Luciferase reporter assay, qRT-PCR, and Western blot) HMGA2, NOTCH1, NOTCH2, NOTCH3, NOTCH4 (qRT-PCR)
**miR-34a-5p** (-)	qRT-PCR	Glioma	U251, A172, and SHG-44	NA	Proliferation Cell cycle Apoptosis Migration Invasión	SIRT1 (Luciferase reporter assay, Western blot, and qRT-PCR)
**miR-34a-5p** (-)	q RT-PCR	Hepatocellular carcinoma	HepG2	NA	Proliferation Cell cycle Apoptosis Migration Invasion	CDK4, CDC2, Cyclin D1, Cyclin A, HSP90B1, HSP27 (Western Blot)
**miR-34a-5p (-)** miR-34b-5p (-) miR-34c-5p (-)	qRT-PCR	Osteosarcoma	U2OS and SAOS-2	Primary tumor samples	Cell cycle Apoptosis	CDK6 and Bcl-2 (Western Blott), E2F3 and Cyclin E2 (Luciferase reporter assay, Western Blot)
miR-34a-5p (-) miR-34b-5p (-) miR-34c-5p (-)	qRT-PCR *In situ* hybridization	Ovarian cancer	SKOV-3	Stage tumors I, II, III, IV	Migration Invasion	MET (qRT-PCR, Western Blotting, *In situ* hybridization) CDK4 (Western blotting).
**miR-34a-5p (-)** **miR-34b-5p (-)** **miR-34c-5p (-)**	qRT-PCR	Pancreatic Cancer	MiaPaCa2 and BxPC3	NA	Cell cycle Apoptosis Tumorsphere formation Tumor Growth in mice	BCL-2 (Luciferase reporter assay, qRT-PCR, Western blotting) NOTCH1 and NOTCH2, (Western blot and qRT-PCR) NOTCH3 and cMet (qRT-PCR) Mcl-1 and Bcl-xL (Western blot)
**miR-34a-5p (-)**	qRT-PCR	Prostate cancer	PC3	NA	Cell cycle Chemoresistance	SIRT1 (qRT-PCR, Western blotting, Luciferase reporter assay) CDK6, E2F3, E2F1, Cyclin D1, BCL2 (Western blot)
miR-34a-5p (-) **miR-34b-5p (-)** **miR-34c-5p (-)**	qRT-PCR	Lung cancer	SCLC cell lines [NCI-H1048 (H1048), SBC5, HCC33, NCIH211 (H211), NCI-H524 (H524), NCI-H841 (H841), NCI-H1688	SCLC, NSCLC resected tumors, and malignant pleural effusions in SCLC patients	Anchorage independant growth, Migration Invasion	c-MET and CDK6 (Western blot)

			(H1688), NCI-H1870 (H1870), NCI-H2141 (H2141), NCI-H82 (H82), NCI-H249 (H249)] and 14 NSCLC cell lines [PC-9, HCC827, NCIH1975 (H1975), NCI-H3255 (H3255), A549, NCI-H1395 (H1395), NCI-H522 (H522), NCI-H838 (H838), HCC15, NCI-H125 (H125), NCI-H460 (H460), NCI-H661 (H661), NCI-H1299 (H1299), and NCIH358 (H358)]			
**miR-34a-5p** (-)	qRT-PCR	Lung cancer	A549, BJ, NCI-H460, Calu-3, NCI-H596, NCI-H1650, HCC2935, SW-900, NCI-H226, NCIH522, NCI-H1299, Wi-38, and TE353	FFPE lung tumor samples, NSCLC tumor samples.	Tumor growth in mice and Proliferation	Ki-67, Caspase-3, c-Met, Cdk4, Bcl-2 (immunohistochemistry)
**miR-34a-5p** (-)<br>miR-34b-5p (-)<br>miR-34c-5p (-)	qRT-PCR	Retinoblastoma	Y79 and Weri-Rb1	NA	Cell viability<br>Cell cycle<br>Apoptosis	*CCND1, CNNE2, CDK4, E2F3, MDMX, SIRT1, EMP1, MLLT3, SLC30A3, CTNND2, PLCG1, ACTR1A, CDC25A, ACTR1A, CDC25A, EFNB1, KCNH2, PPP1R10 (+), LYST (+)* (qRT-PCR)
**miR-34a-5p** (-)	qRT-PCR	Colon cancer	HEK293T and RKO	Primary human colon cancer tissues	Cell adhesion<br>Proliferation<br>Migration<br>Invasion	Fra-1 (Luciferase reporter assay, Western blot, qRT-PCR)<br>MPP-1, MPP-9, and c-MET (Western blot, qRT-PCR)

**miR-34c-5p** (-)	qRT-PCR	Colon cancer	HCT-8	NA	Proliferation Anchorage independent growth, Cell cycle	CDK4, cMyc (Western blot, and qRT-PCR)
**miR-34a-5p** (-) **miR-34b-5p** (-) **miR-34c-5p** (-)	qRT-PCR	Gastric cancer	SC-M1, AGS, AZ521, NUGC-3, KATO III, NCI-N87, and SNU-16	NA	Cell cycle Tumorsphere formation Migration Invasion Tumor Growth in mice	YY1 (Luciferase reporter assay, qRT-PCR, western blot), Plakoglobin (+, 34a, 34b, 34c) E-cadherin (34b, 34c), N-cadherin, Vimentin
**miR-34a-5p** (-)	qRT-PCR, microarray analysis	Glioma	NIH 3T3 and TS543	NA	Proliferation Cell cycle Apoptosis Tumor Growth in mice	PDGFRA (Luciferase reporter assay, Western blot), NOTCH1 (Western blot)
**miR-34c-5p** (-)	qRT-PCR,	Leukemia	KG-1a, THP-I and KASUMI-1	NA	Senescence Apoptosis Cell cycle	E2F3, CDK4, c-Met, c-Myc, cyclin E2, (Western blotting,)
**miR-34a-5p** (-)	qRT-PCR,	Esophageal squamous cell carcinoma	HEK293, EC9706, and TE-1	ESCC tissues	Migration Invasion	MMP-2, MMP-9, FNDC3B (Luciferase reporter assay, Western blotting, and RT-PCR)
**miR-34a-5p** (-)	qRT-PCR,	Acute Myeloid Leukemia	THP-1 and HS-5	NA	Apoptosis	Bax (+), BCL2, Cyt C (+), Atg5. LC-3I, LC-3II (Western blotting), HMGB1 (Luciferase reporter assay, Western blotting, RT-PCR)

Table S1. Validated targets of miR-34 family members. miRNAs in bold were used to elucidated mRNA targets.
